# Mental health impacts of anti-trans stigma on transgender people in Australia: a mixed-methods study

**DOI:** 10.3389/fpubh.2026.1906134

**Published:** 2026-07-30

**Authors:** Sav Zwickl, Sasha Bailey, Rin Chan, Shalem Y. Leemaqz, Joel Murray, Stevie Lane, Foster Skewis, Julian Grace, Eli Ward-Smith, Tomi Ruggles, Ada S. Cheung

**Affiliations:** 1 Department of Medicine, Trans Health Research Group, The University of Melbourne, Naarm/Melbourne, VIC, Australia; 2 Department of Anatomy and Physiology, The University of Melbourne, Naarm/Melbourne, VIC, Australia; 3 College of Medicine and Public Health, Flinders University, Tarndanya/Adelaide, SA, Australia; 4 Neophile, Wiradjuri/Albury, NSW, Australia; 5 Access and Equity, Edith Cowan University, Boorloo/Perth, WA, Australia

**Keywords:** anti-trans, discrimination, gender diverse, media, mental health, stigma, transgender, violence

## Abstract

**Background:**

Anti-trans stigma has escalated globally, yet there is limited empirical evidence examining its mental health impacts on trans populations.

**Methods:**

In a May 2024 mixed-methods cross-sectional online survey, trans people aged ≥16 years living in Australia reported the frequency of seven anti-trans stigma experiences in the previous month, including exposure to anti-trans remarks in the news media or online; personal experience of online bullying, harassment or threats; having thought about, seen or heard about anti-trans rhetoric by Australian politicians and high-profile individuals; or anti-trans rallies; or police violence; or international anti-trans legislation and violence. Generalized linear models examined associations between multiple times weekly exposure to each stigma type and anxiety (GAD-7), depression (PHQ-9), self-harm, and suicidality. Inductive thematic analysis was applied to free-text responses.

**Results:**

Among 807 respondents, multiple times weekly exposure to all seven forms of anti-trans stigma was associated with a statistically higher prevalence of anxiety, depression, self-harm ideation, and suicide ideation (relative risk (RRs) ranging 1.14 to 2.10). Six stigma types were associated with increased risk of suicide planning (RRs 1.41 to 2.55), and five with increased risk of self-harm (RRs 1.32 to 2.07). Qualitative analysis identified three themes: (1) Navigating exposure to news and social media, (2) Experienced and anticipated anti-trans stigma, and (3) Escalating and inescapable anti-trans stigma.

**Conclusion:**

Australian trans communities are frequently exposed to anti-trans stigma, with more frequent exposure associated with a statistically higher prevalence of adverse mental health outcomes. Urgent collaborative responses are required from governments and media platforms to reduce stigma-related harm.

## 1 Introduction

In recent years, there has been a marked escalation in anti-trans stigma; the labelling, stereotyping, and exclusion of trans and gender diverse (henceforth ‘trans’) people by institutions and individuals in ways that restrict access to social, economic, and political power (1, 2). Anti-trans stigma manifests at several levels; through societal norms and institutional policies (structural stigma), direct experiences such as verbal harassment or physical violence (enacted stigma), and the expectations and internalized beliefs trans people hold about how they will be treated (perceived or anticipated stigma) (1).

Anti-trans rhetoric, policies, and violence, as forms of structural and enacted stigma, are closely linked over time, forming what has been termed a ‘trifecta of violence’ (3, 4). While trans people have long been subject to medical and social pathologization, recent media and political discourse is increasingly characterized by misinformation (inaccurate information) and disinformation (deceptive information) about trans people and their healthcare (5). This shift has contributed to policy developments in Western contexts including the United States of America (USA), United Kingdom (UK), and Australia, that restrict access to healthcare, sports participation, single-sex spaces, and legal gender recognition for trans people.

Gender-affirming care, including for children and adolescents who seek it, is supported by major medical organizations (6–8) and is associated with improved mental health and wellbeing, and very low rates of regret and reidentification with sex recorded at birth (9–11). Despite this evidence, mis/disinformation has dominated public debate, contributing to bans on reversible puberty suppression medications (‘puberty blockers’) for trans children and adolescents in multiple jurisdictions despite their continued use for cisgender (cis) children with precocious puberty (12, 13). In the USA, legislative efforts targeting trans healthcare, education, and legal recognition have increased sharply, from 143 bills introduced in 2021 to over 1,000 in 2025, with rising passage rates (14). Similar patterns of anti-trans media and political rhetoric, policies, and violence (collectively referred to as *anti-trans stigma*) have been reported across multiple countries, including Australia, where a national survey found that 80% of respondents perceived a cumulative increase over time, including anti-trans rallies and reported incidents of police violence against trans people (15).

From a public health perspective, anti-trans stigma functions as a social determinant of health, operating across structural, interpersonal and psychological levels to shape health outcomes (1). A growing international literature links exposure to anti-trans stigma with poorer mental health. In the United States, exposure to negative transgender-related media messages has been associated with increased psychological distress (16), and anti-trans ballot measures and legislative debates have been associated with elevated distress among trans people and other stigmatized groups (17, 18). However, much of this evidence concerns discrete political events rather than the chronic, ambient exposure to anti-trans rhetoric that now characterizes the news and social media environment, and most studies are concentrated in the United States. The mental health impact of routine, repeated exposure to anti-trans rhetoric, policy, and violence, including international developments reported in domestic media, remains poorly characterized, particularly in Australia. Existing Australian work has documented rising stigma (15) but has not quantified its association with mental health outcomes.

Our study addresses this gap using a national mixed-methods design among trans people aged 16 years and older living in Australia. We examined the frequency of recent exposure to anti-trans stigma, changes in engagement with news and social media to reduce such exposure, and associations between frequent exposure and mental health outcomes. Qualitative free-text responses were analyzed to further explore lived experiences of anti-trans rhetoric, policy, and violence. We hypothesized that a substantial proportion of participants would report frequent recent exposure to anti-trans stigma and deliberate reductions in news and social media use. We further hypothesized that frequent exposure would be associated with higher likelihood of anxiety, depression, self-harm ideation and behavior, and suicidal ideation, planning, and attempts.

## 2 Materials and methods

### 2.1 Study design and participants

Data were collected as part of *TRANSform,* an ongoing longitudinal study launched in May 2020, open to trans people aged 16 or older living in Australia. Most participant recruitment occurs through social media, with posts shared widely by trans organizations and support groups. Additional recruitment occurs through the use of promotional flyers and posters in some clinics known to provide gender-affirming care, and at in-person LGBTIQA+ events.

Participants first complete an enrolment survey collecting basic demographic data and an email address, which is used to invite them to complete sub-studies annually. One such sub-study was an online survey focused on mental health and exposure to anti-trans rhetoric, policies, and violence. This survey was open to the *TRANSform* cohort between 1 May and 31 May 2024. Data from this survey forms the basis of the present analysis.

*TRANSform* is a trans-led research project. Survey design was led by a group of trans researchers with support from cis clinicians and researchers with expertise in trans health. REDCap electronic data capture tools hosted at The University of Melbourne were used for survey data collection and management. The study received ethical and governance approval by the Austin Health Human Research Ethics Committee (Reference Number HREC/57155/Austin-2019), ACON Research Ethics Review Committee (Reference Number 2020/03) and the Thorne Harbor Health Community Research Endorsement Panel (Reference Number THH/CREP 20-006).

### 2.2 Demographic measures

As detailed in Supplementary material, demographic variables included age, Aboriginal and/or Torres Strait Islander identity, country of birth, highest level of education, and employment status. While recognizing that gender identity is complex and fluid, respondents were asked to select one category for analysis: man/trans man (herein referred to as *trans man*), woman/trans woman (herein *trans woman*), non-binary or gender diverse (including agender; herein *non-binary*), or a culturally-specific gender identity not captured by other options (herein *culturally-specific gender identity*).

### 2.3 Exposure to anti-trans stigma

To assess recent exposure to anti-trans stigma, respondents were asked about their experiences of seven types of stigma in the previous month. Three measures captured experienced stigma through exposure or direct experience (seen or heard about); how often they had *seen or heard anti-trans remarks in the news media*, *seen or heard anti-trans remarks online*, and *personally experienced anti-trans bullying, harassment or threats online.*

Four measures captured perceived or anticipated stigma (thinking about) and/or experienced stigma (seen or heard about) related to specific content or events; how often they had *thought about, seen or heard about anti-trans rallies in Australia*, *thought about, seen or heard about police violence against trans people in Australia*, *thought about, seen or heard about anti-trans rhetoric by Australian politicians and high-profile people*, and *thought about, seen or heard about international anti-trans legislation and violence.* The fixed response options were never, a few times a month, a few times a week, once daily, and multiple times daily.

### 2.4 News and social media engagement

To assess changes in news and social media engagement, respondents were asked “*In the last 12 months, have you stopped or reduced your engagement with news media to avoid or reduce your exposure to anti-trans content?”* and “*In the last 12 months, have you stopped or reduced your engagement with social media to avoid or reduce your exposure to anti-trans content?*.” The fixed response options were stopped, reduced, thinking about stopping or reducing, and never.

Experiences with anti-trans stigma were further explored through free-text responses to the question, “*Is there anything else you would like to share with us about your experiences of anti-trans news media, social media and recent events*?”

### 2.5 Mental health measures

Anxiety symptoms in the preceding 2 weeks were assessed using the Generalized Anxiety-7 (GAD-7) (19). Respondents rated the frequency of seven anxiety symptoms, from “0” (not experienced) to “3” (experienced nearly every day). Total GAD-7 scores range from 0 to 21, with scores of 5–9 indicating mild, 10–14 moderate, and ≥15 severe anxiety symptoms. Scores ≥10 are 89% sensitive and 82% specific for detecting Generalized Anxiety Disorder (19).

Depressive symptoms in the preceding 2 weeks were assessed using the Patient Health Questionaire-9 (PHQ-9) (20, 21). Items are rated from “0” (not experienced) to “3” (experienced nearly every day) producing total scores ranging from 0 to 27. PHQ-9 scores of 5–9 represent mild, 10–14 moderate, 15–19 moderately severe, and ≥20 severe depressive symptoms. PHQ-9 scores ≥10 are 88% sensitive and 85% specific for detecting clinically significant major depression (22).

Self-harm and suicidality in the preceding 12 months were assessed by asking whether respondents had thought about hurting themselves on purpose in some way (*self-harm ideation*), hurt themselves on purpose in some way (*self-harm*), seriously considered attempting suicide (*suicidal ideation*), made a plan about how they would attempt suicide (*suicide planning*), or actually attempted suicide (*suicide attempt*).

### 2.6 Statistical analysis

Descriptive statistics are presented as frequency (percentage) for categorical variables, and median (interquartile range), or mean (standard deviation) as appropriate. Associations between frequent anti-trans stigma and mental health outcomes were examined using generalized linear models with a log-binomial link. Poisson regression with robust standard errors were used where log-binomial models failed to converge. All models were adjusted for age and gender (trans man, trans woman, and other). Relative risks (RRs) were reported rather than odds ratios, as odds ratios can overestimate associations when outcomes are common (23). Analyses were conducted in SPSS and R v4.2.1 (R Foundation for Statistical Computing, Vienna, Austria).

### 2.7 Qualitative analysis

Free-text responses were imported into NVivo v14 (QSR International Pty Ltd., Doncaster, Australia). Inductive thematic methods were conducted as outlined by Braun and Clarke’s (24) six-phase method. An inductive approach was chosen because the study sought to centre respondents’ own framings of their experiences rather than impose pre-existing theoretical categories, consistent with the exploratory, community-led aims of the study and a social constructionist epistemology. This approach offered a structured yet flexible framework for identifying, analysing, and interpreting patterns of meaning in the data (24). One researcher coded the free-text responses one-by-one. Codes were not predetermined prior to commencing coding, were developed inductively, and coding decisions strived to use language from respondent quotes themselves to stay immersed in the data. A second researcher reviewed the coded dataset, and discrepancies and queries with the coding were discussed and resolved. The codes were then subsequently grouped into sub-themes and overarching themes.

Coding was undertaken by researchers with trans lived experience. Consistent with thematic analysis (24), reflexivity is a cornerstone of this analysis process, whereby the researchers actively engage in interpretation, resulting in overarching themes that capture the richness, complexity, and depth of the phenomenon being studied (25). Aligning with calls for “a trans-led future for the field of trans health” (26), and the strengths of “insider” research (27), we treated this positionality as an analytic strength supporting contextually grounded and culturally safe interpretation, rather than as a source of bias requiring an external ‘objective’ coder. We acknowledge that researchers without lived experience may foreground different readings, and that reflexive engagement with positionality is an ongoing consideration in community-led research.

## 3 Results

### 3.1 Sample demographics

Of the 807 respondents, age ranged from 16 to 79 years, mean age was 36 years (SD = 13.2), 286 (35.6%) were trans women, 242 (30.1%) were trans men, 271 (33.7%) were non-binary, and 5 (0.6%) had a culturally-specific gender identity. Thirty-three (4.1%) respondents were Aboriginal and/or Torres Strait Islander. Most respondents were born in Australia (*n* = 681, 84.4%), lived in a metropolitan area (*n* = 658, 81.5%), and had a tertiary qualification (*n* = 655, 81.4%; Table 1).

**Table 1:** Sample demographics.

Demographic variable	*n* (%)
Age (years) (*n* = 807)	
16–25	184 (22.8)
26–35	283 (35.1)
36–45	167 (20.7)
46–55	85 (10.5)
56–65	57 (7.1)
66+	31 (3.8)
Gender identity (*n* = 804)	
Trans man	242 (30.1)
Trans woman	286 (35.6)
Non-binary or gender diverse	271 (33.7)
Culturally-specific identity	5 (0.6)
Aboriginal and/or Torres Strait Islander identity (*n* = 807)	
Aboriginal	30 (3.7)
Torres Strait Islander	0 (0.0)
Aboriginal and Torres Strait Islander	3 (0.4)
Non-Indigenous	764 (94.7)
Unsure	5 (0.6)
Prefer not to say	5 (0.6)
Country of birth (*n* = 807)	
Australia	681 (84.4)
Aotearoa New Zealand	22 (2.7)
Germany	4 (0.5)
Ireland	5 (0.6)
Malaysia	4 (0.5)
Singapore	6 (0.7)
United Kingdom	40 (5.0)
United States of America	14 (1.7)
Other	31 (3.8)
Citizenship status (*n* = 802)	
Australian Citizen	764 (95.1)
Permanent Resident	24 (3.0)
Student Visa	2 (0.2)
Other Temporary Visa	7 (0.9)
Asylum Seeker (SHEV, TPV, another visa)	1 (0.1)
Other	5 (0.6)
Location of residence (*n* = 800)	
Capital city	509 (63.6)
Other metropolitan centre (population ≥ 100,000)	149 (18.6)
Large rural centre (population 25,000–99,000)	70 (8.8)
Small rural centre (population 10,000–24,999)	34 (4.3)
Other rural area (population < 10,000)	15 (1.9)
Remote centre (population ≥ 5,000)	3 (0.4)
Other remote area (population < 5,000)	20 (2.5)
Highest level of education (*n* = 805)	
Secondary or below	150 (18.6)
Non-university tertiary (e.g., TAFE)	183 (22.7)
University—undergraduate	275 (34.2)
University—postgraduate	197 (24.5)
Employment status (*n* = 800) *	
Working—full-time	298 (37.3)
Working—part-time	159 (19.9)
Working—casual	120 (15.0)
Unemployed	119 (14.9)
Pension	90 (11.3)
House duties	39 (4.9)
Volunteer	51 (6.4)
Retired	31 (3.9)
Student—full time	94 (11.8)
Student—part time	73 (9.1)

*Respondents were allowed to select more than one response, so percentages may exceed 100. SHEV—safe haven enterprise visa. TPV—temporary protection visa.

### 3.2 Exposure to anti-trans stigma

Among the three measures of *experienced* anti-trans stigma, 575 (72.1%) respondents reported having seen or heard anti-trans remarks online multiple times weekly, compared to 469 (58.2%) who reported seeing or hearing such remarks in the news media multiple times weekly. Personal experience of anti-trans online bullying, harassment, or threats, multiple times weekly, was reported by 64 (8%) respondents (Table 2).

**Table 2:** Frequency of exposure to anti-trans stigma in the previous month.

Variable	*n* (%)
How often have you seen or heard anti-trans remarks in the news media? (*n* = 805)	
Never	44 (5.5)
A few times a month	292 (36.3)
A few times a week	252 (31.3)
Once daily	68 (8.4)
Multiple times daily	149 (18.5)
Total participants reporting weekly exposure	469 (58.3)
Total participants reporting daily exposure	217 (27.0)
How often have you seen or heard anti-trans remarks online? (*n* = 797)	
Never	32 (4.0)
A few times a month	190 (23.8)
A few times a week	246 (30.9)
Once daily	88 (11.0)
Multiple times daily	241 (30.2)
Total participants reporting weekly exposure	575 (72.2)
Total participants reporting daily exposure	329 (41.3)
How often have you personally experienced anti-trans bullying, harassment or threats online? (*n* = 797)	
Never	577 (72.4)
A few times a month	156 (19.6)
A few times a week	38 (4.8)
Once daily	9 (1.1)
Multiple times daily	17 (2.1)
Total participants reporting weekly exposure	64 (8.0)
Total participants reporting daily exposure	26 (3.3)
How often have you thought about, seen or heard about anti-trans rallies in Australia? (*n* = 802)	
Never	219 (27.3)
A few times a month	479 (59.7)
A few times a week	83 (10.3)
Once daily	10 (1.2)
Multiple times daily	11 (1.4)
Total participants reporting **weekly** exposure	104 (13.0)
Total participants reporting **daily** exposure	21 (2.6)
How often have you thought about, seen or heard about police violence against trans people in Australia? (*n* = 796)	
Never	321 (40.3)
A few times a month	371 (46.6)
A few times a week	76 (9.5)
Once daily	11 (1.4)
Multiple times daily	17 (2.1)
Total participants reporting **weekly** exposure	104 (13.1)
Total participants reporting **daily** exposure	28 (3.5)
How often have you thought about, seen or heard about anti-trans rhetoric by Australian politicians and high-profile people? (*n* = 798)	
Never	83 (10.4)
A few times a month	415 (52.0)
A few times a week	217 (27.2)
Once daily	45 (5.6)
Multiple times daily	38 (4.8)
Total participants reporting **weekly** exposure	300 (37.6)
Total participants reporting **daily** exposure	83 (10.4)
How often have you thought about, seen or heard about international anti-trans legislation and violence? (*n* = 802)	
Never	32 (4.0)
A few times a month	217 (27.1)
A few times a week	288 (35.9)
Once daily	101 (12.6)
Multiple times daily	164 (20.4)
Total participants reporting weekly exposure	553 (69.0)
Total participants reporting daily exposure	265 (33.0)

Of the four measures of *perceived or anticipated* stigma and/or experienced stigma related to specific content or events, having thought, seen or heard about international anti-trans legislation and violence multiple times weekly was most common, reported by 553 (68.9%) respondents. This was followed by having thought, seen or heard about anti-trans rhetoric by Australian politicians and other high-profile individuals (*n* = 300, 37.6%; Table 2).

### 3.3 Reductions in news and social media engagement in the previous 12 months

Stopping or reducing engagement with news media in the previous 12 months to avoid or reduce exposure to anti-trans stigma was reported by 108 (13.5%) and 484 (60.6%) respondents, respectively. Stopping or reducing engagement with social media was reported by 81 (10.1%) and 469 (58.7%) respondents, respectively (Table 3).

**Table 3:** Reductions in news and social media engagement in the previous 12 months.

Variable	*n* (%)
Have you stopped or reduced your engagement with news media to avoid or reduce your exposure to anti-trans content? (*n* = 799)	
Stopped	108 (13.5)
Reduced	484 (60.6)
Thinking about stopping or reducing	106 (13.3)
Never	101 (12.6)
Have you stopped or reduced your engagement with social media to avoid or reduce your exposure to anti-trans content? (*n* = 799)	
Stopped	81 (10.1)
Reduced	469 (58.7)
Thinking about stopping or reducing	121 (15.1)
Never	128 (16.0)

### 3.4 Associations between exposure to anti-trans stigma and mental health outcomes

Nearly half of respondents met criteria for probable anxiety (GAD-7 ≥ 10; *n* = 377, 47.8%), and over half met criteria for probable depression (PHQ-9 ≥ 10; *n* = 450, 58.2%). In the preceding 12 months, 435 (53.9%) respondents reported self-harm ideation, 201 (24.9%) reported self-harm, 207 (25.7%) reported suicidal ideation, 161 (20.0%) reported suicide planning, and 25 (3.1%) reported a suicide attempt (see Supplementary material).

Exposure to all seven forms of anti-trans stigma multiple times per week was associated with statistically higher prevalence of anxiety, depression, self-harm ideation, and suicidal ideation (relative risk (RRs) ranging 1.14 to 2.10), compared with those with less than weekly exposure. Six stigma types were associated with statistically higher prevalence of suicide planning (RRs 1.41 to 2.55), and five with increased risk of self-harm (RRs 1.32 to 2.07; Table 4). Associations were strongest for personal experience of online bullying, harassment, or threats, which was associated with more than double the prevalence of suicidal ideation (RR 2.10, 95% CI 1.58 to 2.78) and suicide planning (RR 2.55, 95% CI 1.87 to 3.49). Associations with suicide attempt were elevated in magnitude but did not reach statistical significance for any exposure, with wide confidence intervals reflecting the small number of respondents who reported attempts (*n* = 25). Exposure to police violence was not significantly associated with self-harm behavior (RR 1.18, 95% CI 0.86 to 1.61), and exposure to international anti-trans legislation and violence was not significantly associated with suicide planning (RR 1.38, 95% CI 0.99 to 1.93).

**Table 4:** Associations between frequency of reported exposure to anti-trans stigma and mental health outcomes.

Exposure variable	Anxiety last 2 weeks RR (95% CI)	Depression last 2 weeks RR (95% CI)	Self-harm ideation last 12 months RR (95% CI)	Self-harm behavior last 12 months RR (95% CI)	Suicidal ideation last 12 months RR (95% CI)	Suicide plan last 12 months RR (95% CI)	Suicide attempt last 12 months RR (95% CI)
Anti-trans remarks in the news media	**1.37 (1.17, 1.60)** ^***^	**1.25 (1.10, 1.42)** ^***^	**1.23 (1.07, 1.40)** ^**^	**1.38 (1.07, 1.78)**	**1.33 (1.03, 1.71)** ^*^	**1.43 (1.06, 1.93)** ^*^	2.11 (0.85, 5.26)
Anti-trans remarks online	**1.60 (1.30, 1.97)** ^***^	**1.47 (1.24, 1.75)** ^***^	**1.30 (1.10, 1.53)** ^**^	**1.85 (1.31, 2.61)** ^***^	**1.68 (1.22, 2.32)** ^**^	**1.71 (1.18, 2.49)** ^**^	2.81 (0.84, 9.38)
Personal experience of anti-trans bullying, harassment or threats online	**1.74 (1.46, 2.08)** ^***^	**1.58 (1.40, 1.79)** ^***^	**1.41 (1.19, 1.66)** ^***^	**2.07 (1.52, 2.81)** ^***^	**2.10 (1.58, 2.78)** ^***^	**2.55 (1.87, 3.49)** ^***^	1.85 (0.64, 5.33)
Thought about, seen or heard about anti-trans rallies in Australia	**1.57 (1.35, 1.84)** ^***^	**1.33 (1.16, 1.51)** ^***^	**1.30 (1.12, 1.51)** ^***^	**1.40 (1.04, 1.88)**	**1.50 (1.13, 1.99)** ^**^	**1.71 (1.24, 2.36)** ^***^	2.06 (0.84, 5.05)
Thought about, seen or heard about police violence against trans people in Australia	**1.32 (1.11, 1.57)** ^***^	**1.26 (1.10, 1.44)** ^***^	**1.18 (1.01, 1.38)** ^*^	1.18 (0.86, 1.61)	**1.42 (1.06, 1.90)** ^*^	**1.68 (1.21, 2.35)** ^**^	1.72 (0.64, 4.58)
Thought about, seen or heard about anti-trans rhetoric by Australian politicians and high-profile people	**1.28 (1.11, 1.48)** ^***^	**1.29 (1.15, 1.45)** ^***^	**1.14 (1.01, 1.29)** ^**^	**1.32 (1.04, 1.68)** ^*^	**1.46 (1.16, 1.85)** ^**^	**1.41 (1.07, 1.85)** ^*^	1.09 (0.49, 2.40)
Thought about, seen or heard about international anti-trans legislation and violence	**1.46 (1.21, 1.75)** ^***^	**1.30 (1.12, 1.51)** ^***^	**1.24 (1.06, 1.44)** ^**^	1.33 (1.00, 1.78)	**1.37 (1.03, 1.83)** ^*^	1.38 (0.99, 1.93)	1.32 (0.53, 3.31)

All models are adjusted for age and gender. RR = risk ratio. Statistically significant results are displayed in bold text with precise degree of statistical significance denoted via superscripted asterixis. Specifically, * denotes *p* < 0.05, ** denotes *p* < 0.01, and *** denotes *p* < 0.001.

## 4 Qualitative themes underlying experiences with anti-trans rhetoric, policies, and violence

In total, 209 participants provided free-text qualitative responses regarding their experiences of anti-trans rhetoric, policies and violence. Responses ranged from 4 to 297 words. Inductive thematic analysis identified three overarching themes: (1) Navigating exposure to news and social media, (2) Experienced and anticipated anti-trans stigma, and (3) Escalating and inescapable anti-trans stigma. Themes and sub-themes are outlined in Table 5.

**Table 5:** Overview of the themes and sub-themes for free-text responses to the survey question “Is there anything else you would like to share with us about your experiences of anti-trans news media, social media and recent events?”

Theme	Sub-themes	Sub-theme summary
1. Navigating exposure to news and social media	1.1 Reduced participation in news and social media	Respondents described stopping or reducing their use of news media and social media to the reduce likelihood of exposure to anti-trans stigma, and association negative mental health impacts.
	1.2 Altered participation in news and social media	Respondents described being proactively selective with what content creators and platforms they used, to curate their news media and social media content.
	1.3 A balancing act	Respondents described trying to balance their needs to be informed of current events and for community connection, with the need to reduce the likelihood of exposure to anti-trans stigma.
2. Experienced and anticipated anti-trans stigma	2.1 Experiences of harassment and violence	Respondents described personal experiences of discrimination and harassment, some of which had flow-on impacts, including to employment opportunities.
	2.2 Anticipating stigma and loss of rights	Respondents described how the prevalence of anti-trans stigma, heightened their anticipation of future stigma and loss of rights in their own lives.
	2.3 Experience of minority stress burden	Respondents described how the prevalence of anti-trans stigma placed them in the position of having to perform labour in their everyday lives, including correcting mis/disinformation and educating others about trans people and rights, as a form of minority stress burden.
3. Escalating and inescapable anti-trans stigma	3.1 Escalation over time	Respondents described a marked escalation in anti-trans stigma over time, particularly in the last decade.
	3.2 Pervasive and inescapable	Respondents described the prevalence of anti-trans stigma as relentless, never-ending, and inescapable.
	3.3 Even allies are sharing it	Respondents described how some of the exposure to anti-trans stigma came from cisgender allies or other trans individuals, who were resharing or commenting on anti-trans content.
	3.4 Cyclical reinforcement	Respondents described news media outlets and social media platforms as key drivers and reinforcers of anti-trans stigma.

### 4.1 Theme 1: Navigating exposure to news and social media

Navigating exposure to news and social media was the most prevalent theme. Many respondents described stopping or reducing their use of news and social media to lower exposure to anti-trans stigma and its mental health impacts.

*“I deleted multiple social media accounts as the anti-trans rhetoric online was causing my significant anxiety issues.” –* trans woman, 40 years

*“I have completely cut myself off all news and social media because of anti-trans rhetoric.” –* trans man, 24 years

*“I largely avoid mainstream news media because it is biased and unhelpful in many ways, including in its representation of anti-trans views.” –* non-binary, 42 years

Others described intentionally altering their online environments by selecting platforms, content creators and privacy settings to reduce exposure.

*“When I am exposed to anti-trans news media, I tend to edit my news source to not show similar content in the future or unfollow the source I accessed the content from in order to stop seeing similar news.” –* trans man, 25 years

*“I left Twitter 12 plus months ago and joined BlueSky and have been able to curate my social media to focus much more on topics I enjoy and when trans topics are discussed it's from trans people, both of which have significantly improved my online experience.” –* trans woman, 34 years

*“I use social media in a limited way - to connect with community - and navigate carefully around privacy and avoiding anti-trans messaging.” –* non-binary, 42 years

Many respondents described this navigation as a balancing act between staying informed, maintaining community connection, and protecting their mental health.

*“I feel torn between a need to reduce exposure to these things and a need to be informed and aware.” –* non-binary, 28 years

*“I would have liked to stay on Facebook in order to access trans supportive groups… and individual people, but completely lost patience with the amount of unhelpful and frankly harmful content I was exposed to… I'm not part of any local trans groups, so I do miss the online ones.” –* trans man, 56 years

*“It is almost impossible to avoid this media and still stay connected to any part of the trans community.” –* non-binary, 27 years

*“The current media climate against trans people is unduly affecting my mental health, however I feel like I need to know the current state of things to keep myself as safe as possible.” –* non-binary, 36 years

### 4.2 Theme 2: Experienced and anticipated anti-trans stigma

Respondents described concrete impacts of public discourse on their daily lives. Some reported that anti-trans harassment affected their online content creation, and employment opportunities.

*“I have been live streaming singing and making music for the past few months and experiencing a lot of negative interactions, there is positive ones too, but a lot of my time is filtering comments and blocking words and asking good people to report bad people. It takes away from what I'm trying to do musically.” –* trans woman, 42 years

*“My football team, which has been a source of safety and community, has been targeted by anti-trans hate groups online. This has led to… trans people on my team being exposed and all of us having to either remove social media accounts or change names on them to avoid further exposure. Personally, this is limiting my opportunities for networking on LinkedIn, putting me at a disadvantage compared to my cisgender peers.” –* trans woman, 27 years

Others described heightened anticipation of stigma or loss of rights.

*“A barrier to my coming out was a fear that my workplace would receive harassment if they didn't fire me, and that even supportive coworkers might come to resent me as a result. This fear was based on anti-trans news media and even pro-trans news media reporting on anti-trans rhetoric and violence.” –* trans man, 29 years

*“I'm terrified of having my rights to healthcare taken away and every time I read about countries in Europe or states/provinces in the Americas restricting access to hormones it brings back [post-traumatic stress disorder] to being forced to go through male puberty as a teenager which is extremely distressing and makes me suicidal.” –* trans woman, 30 years

Some respondents also described the labour involved in correcting mis/disinformation and educating others, as a form of minority stress burden.

*“I find myself constantly trying to educate others… often in discussions where cis women express transphobic views. It can be difficult in the workplace and social settings.” –* trans man, 37 years

*“On a daily basis, often multiple times daily, I am asked… to posit my opinion on a variety of trans issues. The fatigue of constantly speaking to and hearing about such issues is a toll of its own.” –* trans woman, 36 years

### 4.3 Theme 3: Escalating and inescapable anti-trans stigma

Anti-trans stigma was consistently described as escalating over time.

*“I do come across random transphobia a lot more often now than when I came out 15 years ago, online and in person.” –* trans woman, 40 years

*“There has been an increase in anti-trans media coverage and an increase in transphobic bullying on social media platforms over the last 5 years.” –* trans man, 33 years

Stigma was also described as relentless, omnipresent, and inescapable.

*“[It] feels like the walls are closing in on us, that violence and the removal of our basic human rights are ever increasingly a threat (and a reality).” –* trans man, 31 years

*“The anti-trans rhetoric feels overwhelming and never ending.” –* trans man, 45 years

*“It’s difficult to go full remote silence on social media as these political anti trans motivations affect us. It's all over the world.” –* trans man, 33 years

This inescapability was sometimes complicated by cis allies or other trans community members resharing or commenting on anti-trans content, which became a key source of exposure.

*“Most of the anti-trans content I see is from cis people trying to dunk on transphobes by retweeting or sharing the content and adding their own comments disparaging it. It is inescapable, even from allies.” –* non-binary, 32 years

*“Most of the anti-trans media that I see is shared through trans-positive outlets, when they're making statements to condemn the anti-trans media.” –* trans man, 33 years

Lastly, respondents identified news and social media platforms as key drivers and reinforcers of anti-trans stigma.

*“A lot of it appears to be click-baity and inflammatory reporting, which exacerbates and inflames public opinion.” –* trans man, 32 years

*“The media are complicit in propagating the rhetoric. Social media companies are only interested in engagement and don't care about the toxicity or negativity of that engagement. None of them act against the bigots because that would reduce engagement.” –* trans woman, 47 years

## 5 Discussion

This cross-sectional survey of 807 trans people aged 16 years and older in Australia found that exposure to all seven forms of anti-trans stigma types assessed in this study was common, often occurring multiple times weekly. Reducing or stopping news and social media engagement over the previous 12 months was widespread. All seven anti-trans stigma measures were associated with a statistically higher prevalence of probable anxiety and depression, self-harm ideation, and suicidal ideation, six were associated with a statistically higher prevalence of suicide planning, and five were associated with a statistically higher prevalence of self-harm, even after accounting for age and gender. Qualitative findings further highlighted the pervasive nature of anti-trans stigma on trans individuals, including the need for many survey respondents to navigate a ‘balancing act’ between staying informed and connected to community while trying to minimise psychological harm.

### 5.1 Impacts of and strategies to address anti-trans rhetoric in the news media

Trans people are routinely exposed to anti-trans rhetoric via both traditional and online news media. More than half of respondents reported seeing or hearing anti-trans remarks in the news media multiple times weekly in the preceding month, despite most having stopped or reduced news consumption in the previous 12 months. Frequent exposure was associated with a statistically higher prevalence of probable anxiety and depressive disorders, self-harm ideation, self-harm, suicidal ideation, and suicide planning. While previous studies have linked local media coverage of anti-trans rhetoric and policies to adverse mental health outcomes (16, 17), our findings suggest that international anti-trans developments and their media coverage are also associated with poor mental health among trans people in Australia. A recurring concern was the importation of policies seen in the USA and UK that restrict access to healthcare, sports participation, single-sex spaces, and legal gender recognition for trans people [e.g., 12–14]. In response to growing concern about reporting on gender-diverse communities, the Press Council of Australia released advisory guidelines for the reporting on persons with diverse sexual orientation, gender identity, and innate variations of sex characteristics in 2019 (28). These guidelines emphasize accuracy and minimizing harm, including acknowledging the potential mental health impacts of prejudicial language. However, as industry-funded and non-binding guidance, they lack enforceability. In the absence of meaningful consequences, Australian media outlets have continued to publish anti-trans mis/disinformation, often driven by commercial interests. This is reflected in content analyses showing predominantly negative framing of trans people in Australian media (29).

Given the clear associations between news media exposure and adverse mental health outcomes observed in this study, governments should strengthen media regulatory frameworks to improve accountability for media outlets that promote anti-trans mis/disinformation (15). Alongside regulation, accurate and evidence-based counter-messaging (3), and positive trans representation are important strategies, with prior research demonstrating reductions in prejudice and improved attitudes toward trans people following affirming media portrayals (30–32).

### 5.2 Impacts of and strategies to address online anti-trans rhetoric, bullying, harassment, and threats

Consistent with international research (33, 34), almost three-quarters of respondents reported seeing or hearing anti-trans remarks online multiple times weekly. This exposure was associated with a statistically higher prevalence of anxiety and depression, as well as recent self-harm ideation, self-harm, suicidal ideation, and suicide planning. Personal experiences of anti-trans online bullying, harassment, or threats were reported less frequently, but were associated with the highest relative risks of adverse mental health outcomes, including a statistically higher prevalence of suicidal ideation and suicide planning. In response, many participants reported stopping or reducing their social media use while others actively curated their online engagement. Qualitative findings highlighted the complexity of these strategies, which aimed to reduce harm while maintaining access to information and community connection. However, reduced engagement may also limit access to trans-affirming online spaces, which play a key role in identity development with relative privacy and anonymity, social support and community connection (35–37).

Addressing online anti-trans harassment and mis/disinformation requires coordinated and decisive action. Urgency is warranted to protect trans people, who comprise approximately 0.9% of the Australian population (38) and who, as demonstrated in this study, experience disproportionately high exposure to online anti-trans rhetoric, harassment, and threats with clear associations with adverse mental health outcomes. Governments must work collaboratively with social media platforms to strengthen policies addressing online abuse, curb the spread of anti-trans mis/disinformation, and disrupt online communication networks between anti-LGBTQIA+ hate groups (15).

### 5.3 Impacts of and strategies to address anti-trans political rhetoric and violence in Australia

In Australia, there was a marked escalation in anti-trans rhetoric coinciding with the national tour of UK-based lobbyist Kellie-Jay Keen-Minshull (also known as Posie Parker) in early 2023, alongside widely reported incidents of police violence against trans people [Melbourne Activist Legal Support (39)]. Although respondents did not refer to specific events and we cannot attribute the observed associations to any single event, our findings may suggest sustained mental health impacts of this period of escalation. More than one in 10 respondents reported thinking about, seeing or hearing about anti-trans rallies or police violence against trans people in the preceding month, and these exposures were associated with a statistically higher prevalence of anxiety, depression, self-harm ideation, suicidal ideation, and suicide planning.

Anti-trans rhetoric by Australian politicians and other high-profile individuals has also increased over the past decade (40, 41), alongside several legislative bills proposed to the Australian Senate to restrict trans rights [e.g., (42, 43)]. More than one-third of respondents reported frequent exposure to such rhetoric, which was associated with a statistically higher prevalence of anxiety, depression, self-harm ideation, self-harm, suicidal ideation, and suicide planning. Since data collection, further escalation has occurred. For example, in early 2025, the Queensland State Government implemented a state-wide ban on reversible puberty suppression medications and gender affirming hormone therapy for trans youth, despite an independent evaluation confirming the safety and evidence-base of existing care and recommending further resource allocation (44, 45). This policy shift exemplifies how anti-trans political rhetoric can influence legislative action with direct implications for health and wellbeing, aligning closely with the qualitative themes of experienced and anticipated anti-trans stigma and of escalating and inescapable anti-trans stigma.

In light of the profound negative mental health impacts of anti-trans political rhetoric, it is imperative that all levels of government work collaboratively with trans communities and human rights organizations to establish clear expectations for political accountability in Australia. There is an urgent need for sustained investment in community-led prevention and education initiatives that combat anti-trans mis/disinformation, promote evidence-based practice, and address transphobia and other forms of discrimination at a structural and societal level. Legislative safeguards are also needed to protect access to evidence-based healthcare that is supported by professional medical bodies and clinical guidelines, and to prevent political interference in clinical decision-making. This is essential not only for gender-affirming hormonal care, but for all areas of healthcare vulnerable to disinformation or politicization.

### 5.4 Limitations

The cross-sectional design and non-probability online sampling limit causal inference and generalisability. Although no *a priori* power calculation was conducted, the sample of 807 provided precise estimates for most associations, as reflected in the confidence intervals reported in Table 4; wider intervals for rarer outcomes (notably suicide attempt) indicate limited precision for those estimates. The sample were relatively young, predominantly born in Australia, holding Australian citizenship and largely metropolitan. These findings cannot be generalized to older trans people, those born overseas, those without Australian citizenship, and those living in regional and remote areas, who may have limited access to news media and social media, and/or may experience different forms of stigma. Beyond Aboriginal and/or Torres Strait Islander identity (4.1% of the sample) and country of birth, ethnicity was not recorded. We are therefore unable to determine the ethnic and cultural composition of the sample or to examine whether mental health outcomes or stigma exposure differed across ethnic groups. Given Australia’s cultural and linguistic diversity, future studies should collect detailed ethnicity data and target harder to reach trans populations, to identify groups facing compounded or intersectional stigma. As an Australian sample exposed to a specific national media, political, and policy environment, findings may not transfer to trans populations in other countries with different sociolegal contexts.

No standardized definition of ‘anti-trans’ was provided, allowing for varied interpretations that could have impacted responses. Measures capturing whether respondents had “thought about, seen or heard about” exposures did not distinguish between perceived or anticipated stigma and direct exposure, nor between mediated and personal experiences. In addition, exposure measures referred to the previous month, while self-harm and suicidality were assessed over the previous 12 months, precluding temporal ordering. Associations should therefore be interpreted with caution. The PHQ-9 and GAD-7 are widely used screening tools but have not been formally validated in trans populations, and their items may not fully capture the ways distress is experienced or expressed by trans people. This highlights the need for validated, population-appropriate measures in this population that is known to experience anxiety and depression at rates considerably higher than the general population (46, 47), and our prevalence estimates should be interpreted with this caveat. Additionally, several topics and events were not examined, including religious anti-discrimination debates, legal gender recognition reforms, conversion practices, census inclusion, sporting policies, and access to gender affirming care. Further research should examine these exposures and their mental health impacts.

## 6 Conclusion

Trans people in Australia frequently think about, see and hear anti-trans rhetoric, policies, and violence and these experiences are associated with a statistically higher prevalence of adverse mental health outcomes. An immediate multi-pronged response is needed to combat the global escalation in anti-trans stigma. This must include collaborative action between governments, news media outlets, and social media platforms, alongside funding for research to understand and address the drivers and impacts of anti-trans rhetoric, policies and violence.

## Data Availability

The datasets presented in this article are not readily available because requests must be assessed to ensure that the related research is deemed to be of benefit to the trans and gender diverse community. Reasonable requests will undergone Austin Health Human Research Ethics Committee approval in the form of an amendment. Requests to access the datasets should be directed to SZ, sav.zwickl@unimelb.edu.au.
