# Supplementary material for: Mental health impacts of anti-trans stigma on transgender people in Australia: a mixed-methods study
Source: Front Public Health. 2026 Jul 30;14:1906134. doi: 10.3389/fpubh.2026.1906134 (PMC13468911; doi:10.3389/fpubh.2026.1906134)
Supplement: Supplementary file 1 [file Table_1.docx]

Supplementary Material

# 1 Supplementary Table 1. Survey items relevant to this study.

| **Question and Instructions** | **Response options** |
| --- | --- |
| What is your date of birth? | [calendar] |
| What country were you born in?  **select one* | 1, Australia  2, New Zealand  3, Other (free-text) |
| Do you identify as Aboriginal or Torres Strait Islander? | 1, Yes, Aboriginal  2, Yes, Torres Strait Islander  3, Yes, both Aboriginal & Torres Strait Islander  4, No  5, Prefer not to say |
| How do you describe your gender?    *We acknowledge that you may have multiple descriptors for gender, or your experience of gender may be fluid, but for research classification purposes please select the option that best reflects your gender.*  **select one* | 1, Man / Trans man  2, Woman / Trans woman  3, Non-binary or gender diverse (including Agender)  4, A culturally specific identity not captured by other available options |
| Where do you currently live?  **select one* | 1, Capital city  2, Other metropolitan centre (population ≥ 100,000)  3, Large rural centre (population 25,000-99,000)  4, Small rural centre (population 10,000-24,999)  5, Other rural area (population < 10,000)  6, Remote centre (population ≥ 5,000)  7, Other remote area (population < 5,000) |
| What is the highest level of education that you have completed?    **select one* | 1, Never attended school  2, Primary or below  3, Secondary or below  4, Non-university tertiary (e.g. TAFE)  5, University - undergraduate  6, University – postgraduate |
| What best describes your current employment status?    **select all that apply* | 1, Working full time  2, Working part time  3, Working casually  4, Unemployed  5, Pensioner  6, House duties  7, Volunteer  8, Retired  9, Student full time  10, Student part time  11, My visa does not allow me to work |
| On average, in the last month, how often have you seen or heard anti-trans remarks in the news media?  **select one* | 1, Never  2, A few times a month  3, A few times a week  4, Once daily  5, Multiple times daily |
| In the last 12 months, have you stopped or reduced your engagement with news media to avoid or reduce your exposure to anti-trans content?  **select one* | 1, Stopped  2, Reduced  3, Thinking about stopping or reducing  4, Never |
| On average, in the last month, how often have you seen or heard anti-trans remarks online?    **select one* | 1, Never  2, A few times a month  3, A few times a week  4, Once daily  5, Multiple times daily |
| On average, in the last month, how often have you personally experienced anti-trans bullying, harassment or threats online? For example, someone leaving a transphobic comment on something you posted on social media.  **select one* | 1, Never  2, A few times a month  3, A few times a week  4, Once daily  5, Multiple times daily |
| In the last 12 months, have you stopped or reduced your engagement with social media to avoid or reduce your exposure to anti-trans content?    **select one* | 1, Stopped  2, Reduced  3, Thinking about stopping or reducing  4, Never |
| On average, in the last month, how often have thought about, seen, or heard something about anti-trans rallies in Australia?  **select one* | 1, Never  2, A few times a month  3, A few times a week  4, Once daily  5, Multiple times daily |
| On average, in the last month, how often have thought about, seen, or heard something about police violence against trans people in Australia?  **select one* | 1, Never  2, A few times a month  3, A few times a week  4, Once daily  5, Multiple times daily |
| On average, in the last month, how often have thought about, seen, or heard something about anti-trans rhetoric by Australian politicians and high-profile people?  **select one* | 1, Never  2, A few times a month  3, A few times a week  4, Once daily  5, Multiple times daily |
| On average, in the last month, how often have thought about, seen, or heard something about international anti-trans legislation and violence?  **select one* | 1, Never  2, A few times a month  3, A few times a week  4, Once daily  5, Multiple times daily |
| Is there anything else you would like to share with us about your experiences of anti-trans news media, social media, and recent events? | Free-text |
| Over the last 2 weeks, how often have you been bothered by any of the following problems?    1, Feeling nervous, anxious, or on edge  2, Not being able to stop or control worrying  3, Worrying too much about different things  4, Trouble relaxing  5, Being so restless that it's hard to sit still  6, Becoming easily annoyed or irritable  7, Feeling afraid as if something awful might happen  **For each statement select one response from 'not at all' to 'nearly everyday'.* | 1, Not at all  2, Several days  3, More than half the days  4, Nearly everyday |
| Over the last 2 weeks, how often have you been bothered by any of the following problems?    1, Little interest or pleasure in doing things  2, Feeling down, depressed, or hopeless  3, Trouble falling or staying asleep, or sleeping too much  4, Feeling tired or having little energy  5, Poor appetite or overeating  6, Feeling bad about yourself or that you are a failure or have let yourself or your family down  7, Trouble concentrating on things, such as reading the newspaper or watching television  8, Moving or speaking so slowly that other people could have noticed? Or the opposite - being so fidgety or restless that you have been moving around a lot more than usual  9, Thoughts that you would be better off dead or of hurting yourself in some way  **For each statement select one response from 'not at all' to 'nearly everyday'.* | 1, Not at all  2, Several days  3, More than half the days  4, Nearly everyday |
| Have you experienced any of the following?    1, You thought about hurting yourself on purpose in any way  2, You hurt yourself on purpose in any way  3, You seriously considered attempting suicide  4, You made a plan about how you would attempt suicide  5, You actually attempted suicide  6, You know someone who has died by suicide  7, You provided care and support to a suicidal person    **For each statement, indicate whether you have had this experience in the last 12 months, over 12 months ago or never. If you have had this experience within the last 12 months and over 12 months ago, select both these options.* | 1, Last 12 months  2, Over 12 months ago  3, Never |

# 2 Supplementary Table 2. Rates of anxiety, depression, self-harm and suicidality

| **Variable** | **n (%)** |
| --- | --- |
| Anxiety (GAD-7 total score) (n=789) |  |
| Score 0-4: Minimal Anxiety | 225 (28.8) |
| Score 5-9: Mild Anxiety | 187 (23.7) |
| Score 10-14: Moderate Anxiety | 180 (22.8) |
| Greater than 15: Severe Anxiety | 197 (25.0) |
| Depression (PHQ-9 total score) (n=773) |  |
| Score 0-4: None | 127 (16.4) |
| Score 5-9: Mild Depression | 196 (25.4) |
| Score 10-14: Moderate Depression | 164 (21.2) |
| 15 - 19: Moderately Severe Depression | 145 (18.8) |
| 20-27: Severe Depression | 141 (18.2) |
| Thought about hurting self on purpose in any way in last 12 months (n=807) |  |
| Yes | 435 (53.9) |
| No | 372 (46.1) |
| Hurt self on purpose in last 12 months (n=807) |  |
| Yes | 201 (24.9) |
| No | 606 (75.1) |
| Seriously considered attempting suicide in last 12 months (n=807) |  |
| Yes | 207 (25.7) |
| No | 600 (74.3) |
| Made a plan about how would attempt suicide in last 12 months (n=807) |  |
| Yes | 161 (20.0) |
| No | 646 (80.0) |
| Attempted suicide in last 12 months (n=807) |  |
| Yes | 25 (3.1) |
| No | 782 (96.9) |
